# Routine to Rare Risk - A Case Study of Firecracker Explosion Disaster in India

**DOI:** 10.1371/currents.dis.d5a27e47c8decbd4263813a15c47e07e

**Published:** 2018-09-27

**Authors:** Faisel T Illiyas, Shibu K Mani

**Affiliations:** Jamsetji Tata School of Disaster Studies, Tata Institute of Social Sciences, Mumbai, Maharashtra, India; Jamsetji Tata School of Disaster Studies, Tata Institute of Social Sciences, Mumbai, Maharashtra, India

## Abstract

**Introduction:**

In India quite a few religious festivals and cultural fairs are accompanied by public display of fireworks. The grandeur of the festivals is often evaluated by the common man with respect to its colorful firework displays. Firecracker accidents during mass fireworks at public display venues may be disastrous in its consequences and damages. A fire cracker disaster which occurred during a religious public firework display event at Puttingal in Kerala, India was documented and analyzed to figure out the safety concerns and good practices, towards making a reference for effective emergency management.

**Methods:**

The fire cracker incident was studied on the broader perspective of disaster management. Inputs from agencies involved in emergency response, casualty management, damage assessment and general administration as well as the perspective of victims and the public who witnessed the event was incorporated in to the study through participatory observation, field visits and face to face discussions.

**Result:**

The response followed by the firecracker explosion was analyzed in three phases based on the time frame of response. Influence of traditions and culture in firework organization, the mandatory legal requirements for firework displays and the current safety practices followed were evaluated in the background of this rare firecracker risk which turned out to be a major disaster in the state of Kerala in India.

**Conclusion:**

Public display of fireworks in Puttingal temple was organised despite of the legal permission from competitive authority. Negligence of law, non-sensitivity of public towards fire work safety, competitive nature of event organizers and social pressure from religious groups traversed the basic fire work safety requirements, ultimately triggered the largest fire cracker disaster in Kerala.

## Introduction

Firecrackers are used worldwide to celebrate popular social events, mass gatherings and religious festivals. The hazardous fireworks industry in India has apparently been nurtured on traditional, cultural and religious constructs. Fire crackers are perceived as inevitable part of celebrations in many festivals in India. During the festival seasons, firecrackers are available in every nook and corner of the country and people light the firecrackers at courtyards, roads and other public places. In organized social events, the organizers stage large public firework displays by engaging persons who had handled such events in the past or contractors. Ocular injuries related to firecracker usage are reported during each festival season in India^1^ bringing it into a category of routine risk. Accidental explosion and widespread human and economic loss have been reported occasionally from manufacturing units, but few fatal incidents were reported from the display grounds. Sekar et al^2^ listed 9896 accidents in fireworks and match works industries from ‘Sivakasi’ (the firecracker hub of India located in Tamil Nadu state of India) for the 2003 -2010 period, of which 398 were reported as fatal incidents.

Kerala, the southwestern coastal state of India, is rich in religious and cultural diversity. Traditional fireworks at public places or in the premises of religious institutions are major attraction of festivals in Kerala. As part of the annual festivals, many temples and churches conduct fireworks, with duration ranging between thirty minutes to five hours. This has become a regular cultural practice for various entities within the society. Major festivals which are renowned for public display of fireworks use hundreds to thousands of kilograms of gun powder. Fireworks at limited infrastructure facilities surrounded by crowded precincts can be seen across the state. Indiscriminate use of fireworks in very small open space or densely populated areas has high probability of injuries and fatality rate if an incident is triggered.

Fire crackers stored for the public display of fireworks at Puttingal Goddess Temple, Kollam district of Kerala, India exploded in the early morning of 10th April 2016. Documenting the sequence of events and doing a preliminary analysis of such incidences have administrative and academic value for creating disaster-free religious festival management practices besides contributing to developing awareness a culture of safety and maintenance. Here we try to describe the event to find the factors which lead to the disastrous event, its consequences, and to explorehow the situation was managed.

## Methods

A case study based qualitative approach has been followed in this study for the in depth inquiry of the fireworks event and subsequent response activities. The first author had involved in the onsite emergency management operations of the Department of Disaster Management, Government of Kerala. A time line of emergency response operations of each stakeholder agencies were documented during the real-time response operations. Purposive sampling method adopted for interviewing concerned officials which covered the district level commanding officers of General Administration, Police, Fire & Rescue, and Health departments. A field survey was carried out in the affected area from 13.04.206 to 15.04.2016 for rapid damage assessment and to document the views of the affected families. The survey was conducted by two teams lead by the authors. The affected area was divided into two zones based on the distance from the explosion point. In Zone I (area falls within 200 meter radius from explosion point), fifty eight buildings including residential houses, public building and small grocery shops were visited and documented the type of damages. In Zone II (area falls within 200 meter to 500 meter radius from the explosion point), twenty two houses which had suffered visible damages were visited for rapid damage assessment. Ten survivors who suffered secondary injuries from the airborne blast debris and another ten survivors who suffered burn injuries from the blast wave were interviewed at the District Hospital, Kollam on 16.04.2016 to obtain the perception of survivors about the incident. The interviews were performed within one week of the firecracker disaster, hence obtaining written consent from the participants was difficult. Before the interview, the participants were briefed about the purpose of the study and taken their verbal consent. All interviews including the consent statements of the participants were recorded using digital voice recorders. The study was approved by the Doctoral Advisory Committee of the first Author as part of his Doctoral Research at Tata Institute of Social Sciences, Mumbai, India.

## The Firecracker Disaster

The Puttingal Goddess temple has a tradition of nearly 500 years and its annual festival is customarily held for a week period generally in the month of April with special prayers, competitive fire works and various cultural events. The festival area is an open ground without a compound wall; hence specific ingress and egress points were not in place. People could reach the venue from all the directions. The festival organizers had outsourced the cracker manufacturing and fireworks display to a licensed contractor belongs to the locality and the fire crackers manufactured by the contractor were used for the competitive fireworks in Puttingal temple. The common crackers manufactured locally and used in the fireworks are of high decibel crackers. The manufactured products were stockpiled in the permanent and temporary storage houses located in the temple ground premises. The permanent store house located on the south side of the temple known as ‘South Kambappura’ was a single storied concrete building, hereafter referred as ‘south store house’. Manufactured crackers from the temporary store houses located on the outskirt of the temple ground were brought to the ‘south store house’. The layout of the incident location is given in figure 1.

**Layout of the of the fire cracker explosion area. A- Temple with compound wall, B-temporary storage unit of manufactured fire cracker products, C- the exploded south firecracker store house and D- Firing point. The area cordoned for firework is bound by the red dotted line. Spectators watched the fireworks from behind this marked line.**
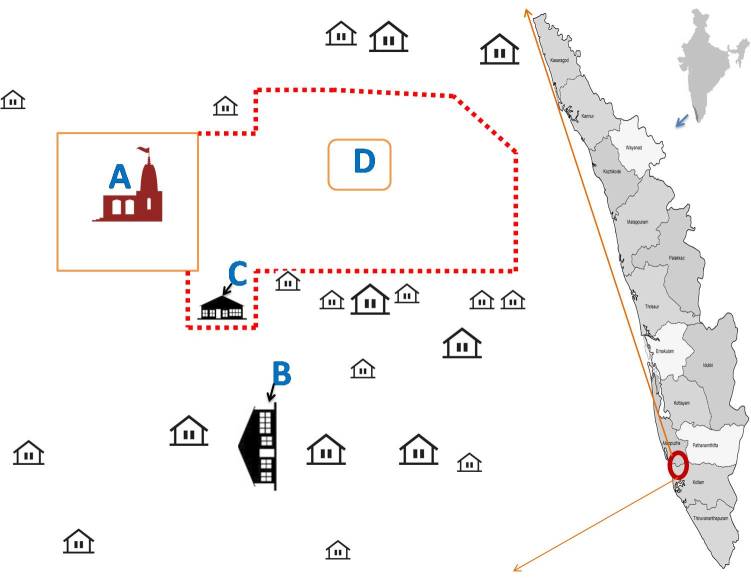

The fire work commenced by 23:45 hours on 09.04.2016 and would have lasted for 4 to 5 hours and would have finished by the next day early morning between 04:00 and 05:00. The firing point was hardly 50 meters away from the south store house. At the time of fireworks, the firing team took the crackers from the south store house and carried it to the firing area, which had anchored iron mortars for consecutive firing (Figure 2). The area demarcated by red line was cordoned off by rope barricades and spectators were allowed to watch the fireworks from the rear of the barricades. The crowd was made up of families, women, children and elderly people. To enjoy a better view of the firework display, several families watched the event from the 1st floor or terrace of buildings around the display ground. The fireworks lead by two groups progressed in a competitive manner, and was interrupted by a short interval by 02:45. A majority of the spectators’, particularly family groups, left the temple premises during the interval time; had it been otherwise, the casualty would have been higher.

**Iron mortars anchored at the firing area of Puttingal temple. Locally crafted gun powder mixes are filled into the half buried mortars and fired for display. The Explosive Rules, (2008) specifies that mortars of the same size and shape shall be grouped and spaced at least 50 centimeters apart. Minimum of 10 meters shall be maintained between groups of different sized mortars. At Puttingal temple, mortars of different sizes were used together without maintaining the specified safety distances.**
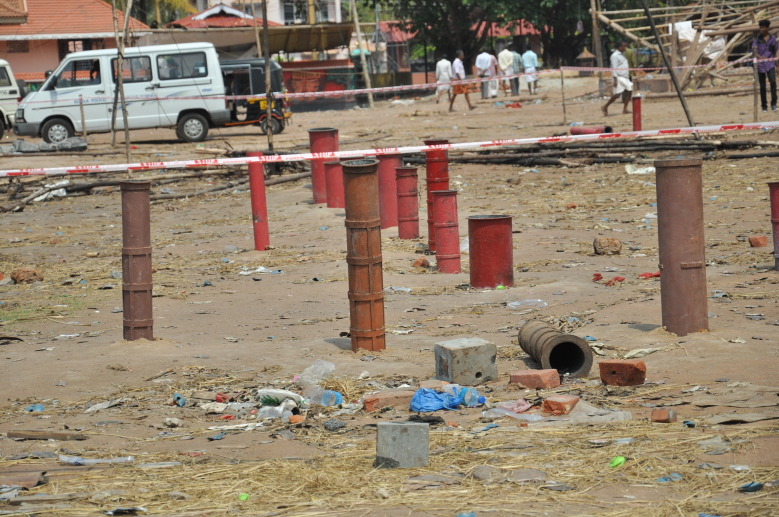

When the firework was resumed after 10 minutes, a section of the firework lovers breached the cordoning and occupied positions very close to the firing point and store house. Crackers emitting very high intensity sounds and wider colorful displays fired continuously, building up to a grand finish to the firework display. At the fag end of the firework display by around 03:10 hours on 10th April 2016, a major light and sound emitting cracker burst at a height of less than 100 meter from the ground and the resulting fiery sparks fell in and around the south store house. About 70% of the stored fire crackers had already been fired for the display by then. The stockpiled crackers in the south store house caught fire and the end result was a massive explosion. Unfired crackers in the south store house exploded completely in 20 to 30 seconds leading to the worst ever fire cracker disaster in the history of Kerala, or for that matter, even in India. The store house made of concrete was totally devastated and its concrete parts propelled in all the directions. Individuals who were watching the fireworks from the close vicinity of the south storehouse were shoved back roughly by the pressure waves generated by the blast and many of them succumbed to the battering against the temple compound walls or similar hard structures in the surroundings.

Immediately after the explosion, the power supply to the area was completely disrupted leaving the entire area in darkness. The explosion and collapse of the concrete store house engulfed the area with building debris, dust, smoke and smell of gun powder. Radial dispersal of projectiles from the point of explosion was observed. The survivors of the accident recounted that ‘they saw a huge fire and heard bomb-like noises and then there was total darkness’. On regaining consciousness, several survivors thought that they had lost their visibility as they could not see anything around, but just the scream of people.

## Event Impact

The impact of the disaster spread over 1 km radius from the explosion point. The exploded south store house was the locus of the damages. Explosion of the concrete building turned the debris into deadly projectiles that travelled up to 1 kilometer in all the directions (Figure 3 to 5). The devastating explosion killed 109 people 1250 people suffered injuries of various categories. Victims succumbed to severe burn injuries within a 50 meter radius of the exploded storehouse where as critical injuries were inflicted by flying concrete debris within a 100 meter radius. A bike rider lost his life from falling concrete debris at a distance of 700 m from the explosion point. Residential buildings, commercial shops, office buildings, open wells and electrical infrastructure in the area were seriously damaged in a 500 m radius. Houses within a 300 m radius suffered serious structural damages including failure of columns, beams, concrete roof, concrete slabs and brick walls and became un-inhabitable. Collapse of wooden roof structure, damages to thatching tiles, iron sheets, window glasses, wooden doors, fiber doors, furniture, false ceiling etc have occurred within 1 km radius of the explosion. An assessment by the Public Works Department accounted 358 damaged buildings including houses, commercial shops, police station, court, hospital, school, bank, daycare centre and structures owned by the temple. The building infrastructure suffered a loss of 0.3 million USD with substantial damage in allied infrastructures like power and water network.

**(A) A house located at 65 meter distance from the explosion point suffered severe structural damages by flying concrete debris of the exploded storehouse. (B) A portion of the damaged house – view from inside:**
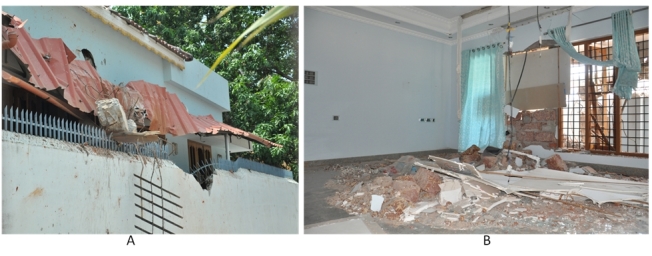

**Concrete pieces of the exploded building found on the terrace of a house. It was like showering of concrete pieces that damaged building roofs covered by clay tiles, tin sheets and asbestos sheets.**
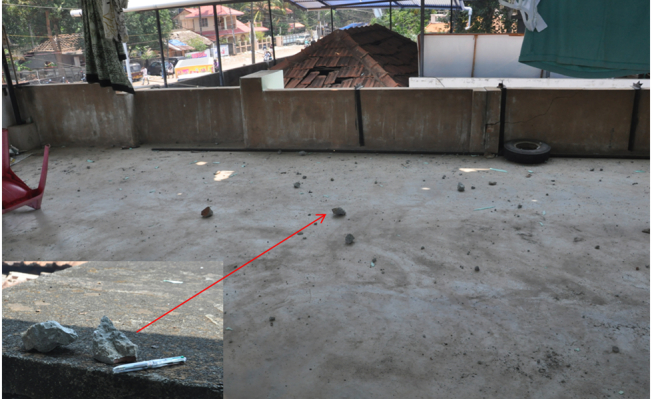

**Flying debris damaged a wall and penetrated into a kitchen of a house. Cracks developed by the impact of the debris projectiles can be seen along the yellow line.**
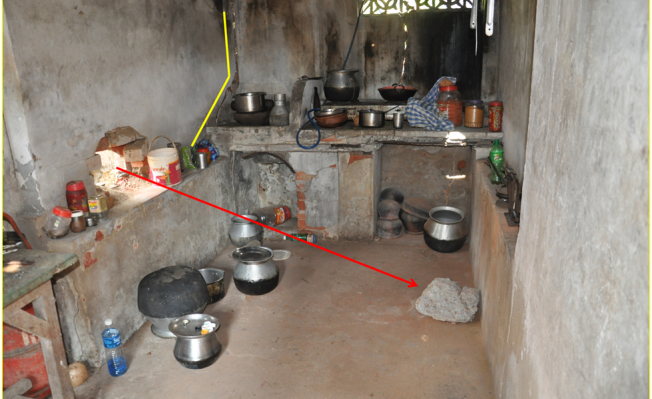

Explosion of the concrete building was the prime reason for majority of casualties. Foreign particles and concrete pieces of various sizes pierced the victim bodies and inflicted severe damages to internal organs. The medical examination reports reveal that severe head injuries, crushing and damages to internal organs became the major reason for death and injuries of above 80 % of the casualties and the remaining cases are purely due to burning.

The response operations and challenges faced by the administration and other stakeholder agencies in the wake of the firecracker explosion in response to the explosion is elucidated as three phases.

## Phase I: Emergency Response

The police and the fire and rescue team stationed near the temple, the spectators and the survivors of the incident became the first responders to the explosion. Emergency response operations set in motion as soon as the blaze of explosion receded. The response operations in the early morning had to be carried out in a complex environment. Disruption of electric supply in the disaster area, presence of un-exploded firecrackers, risk of further explosions, limited availability of skilled human resource for search and rescue, absence of on-site medical facilities, rescue of burned victims, absence of sufficient number of ambulances for medical transportation, congestion of cell phone networks and lack of expertise to defuse the unused crackers hoarded in temporary stores in the periphery of the temple premises were the major challenges faced by the responders.

Headlights of the police vehicles and private vehicles parked near the temple were directed towards the disaster spot and the initial rescue operations were conducted under this sparse light. The rescuers had no clue about the quantity of the exploded fire crackers, whether the entire quantity had exploded or whether there would be another explosion. The fire and rescue team sprayed water over the temporary storage houses and dispersed fire cracker parts to suppress the likelihood of further explosions. Unfired crackers sourced from the scene were kept immersed in water, so that secondary explosions could be averted. Officials and local rescuers pooled their efforts to extricate the injured and the dead bodies from the debris and for transporting them to nearby hospitals.

Locally available private vehicles including taxis, goods carriers and service buses were used for medical transportation in the initial hours followed by ambulances. Majority of the seriously victims were transported to the District Hospital at Kollam (45 minute drive for 23 km distance in normal traffic) and triaging was done there. Besides, a few patients were admitted to private hospitals at Kollam. Seriously injured were transported to Medical College Hospital, Thiruvananthapuram (1.5 hour drive in normal traffic for 53 kilometers from explosion point). Locations of District hospital, Kollam and Medical college hospital, Thruvananthapuram are given in figure 6. More than 100 local people were actively involved in the search and rescue operations till the last injured was transported to the hospital. Free movement of rescue vehicles was ensured by the traffic police by deploying additional traffic management teams under four assistant commissioners. For additional ambulances, drivers were picked from their homes by police and directed to medical transportation.

**Locations of District hospital, Kollam and Medical college hospital, Thiruvananthapuram where majority of casualties were accommodated (Map not to the scale).**
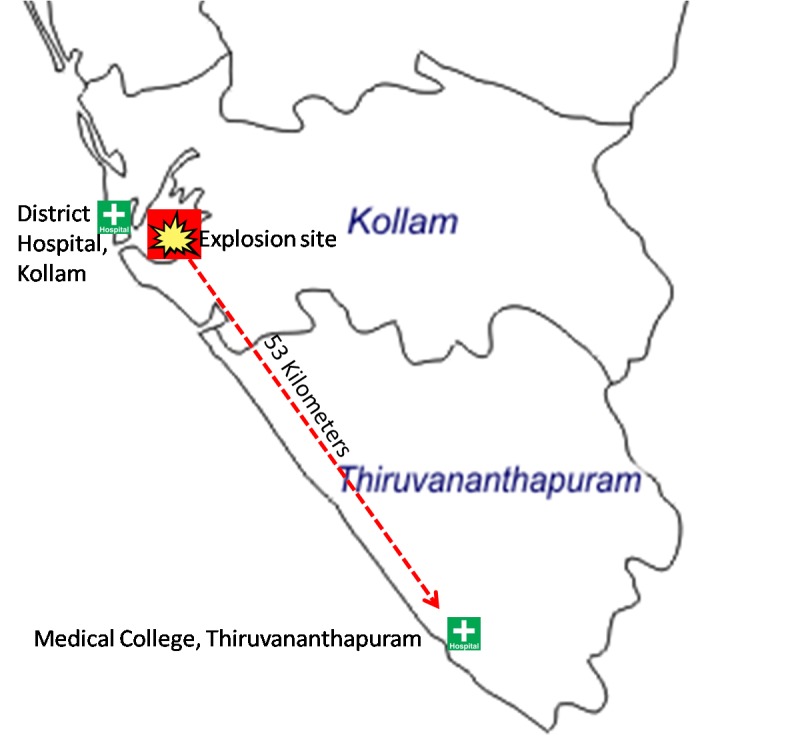

The available means of human expertise and other resources were utilized in the initial hours of response processes till additional resources were made available. An excavator was brought in by 05:00 hours to quicken the rescue operations from under the building debris and rubble. Except the rescue of a victim who had been trapped under the debris of the exploded store house, ninety percentage of the rescue operations could be finished before sun rise. The lacerated body parts from the disaster area packed in sacks by the police and carried to the hospitals before the last victim was rescued and transported by 06:30 hrs. The emergency response agencies could finish the first phase of response, I.e. onsite emergency operations within 3.5 hours of explosion. A timeline indicating the response operations is given in figure 7.

**A schematic representation of operations and time line followed by administration and operational departments.**
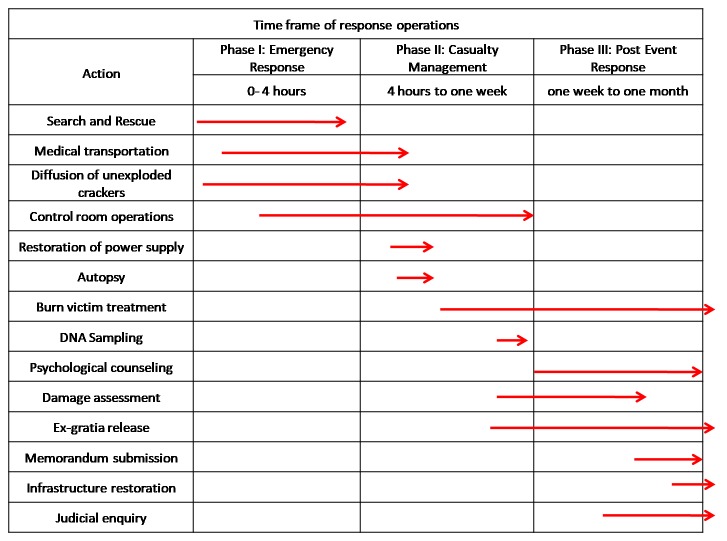

## Phase II: Casualty Management

Mobile networks in the disaster area became ineffective due to heavy traffic; the only means of communication between the response agencies was police wireless network. Onsite police team passed on the message of explosion to district control room and the control room in turn communicated the information to all police stations in Kollam and Trivandrum districts directing available policemen to report at the explosion site. Police from respective stations reached the hospitals in Kollam district and informed the hospital in-charges to prepare for the mass casualty reception. The district collector (head of district administration) and district police chief reached the spot in one hour and coordinated response operations. The event was announced as Level II disaster^3^ requesting support from all stakeholder departments from the State. Doctors from Kollam and Thiruvananthapuram districts were called for mass casualty management, many of them including private practitioners turned to the hospitals without any formal intimation. A control room at disaster spot by police, a district control room by district administration, a state control room by disaster management department and information counters at hospitals were established in two hours. Contact details of control rooms given to the media appeared as scrolls in regional news channels. Police men with wireless communication devices were deployed at the casualty of all referred hospitals to coordinate medical response and casualty transportation.

The incident was reported to National Disaster Response Force and National Disaster Management Authority and the occasion was raised as Level III disaster by 07:00 hours on 11.04.2016. The State requested for two air ambulances from Indian Air Force and two medical teams from Indian Army and the request was addressed by 10:30 hours for medical transportation and expert burn treatment. A special cabinet of the State Government was convened at Kollam by 13:30 hours to review the response, medical care and additional resource requirements. At 14:30 hours, National Disaster Response Force team arrived at the state capital. Since the rescue operations were already over within seven hours, National Disaster Response Force team was kept as stand by at Trivandrum. Doctors from Indian Army, Indian Air Force and Indian Navy were deployed to Hospitals in Kollam district where as doctors from the All India Institute of Medical Sciences and National Disaster Response Force was positioned at Medical College, Trivandrum.

Charred dead bodies and strewn body parts scattered around the exploded storehouse were found mutilated beyond recognition. Identification of dead and missing disaster victims is imperative with respect to its humanitarian, medical and legal aspects like claim on legal heirship and financial compensation for the kith and kin etc. DNA samples of all burned dead bodies and speckled body parts were collected and DNA mapping was carried out for victim identification.

The authors observed a few post-traumatic mental stress cases especially in children during explosion impact mapping field visits. Health department constituted mobile medical teams of specialist doctors, psychiatrists, and counselors to address temporary hearing impairments and post traumatic mental stress suffered by bereaved families and people in the neighborhood of the temple. The medical teams conducted house hold level visits and cluster wise screening camps for case identification. The treatment and counseling continued till the victims were recovered or for a maximum period of one month.

The disaster occurred during the campaigning period of the Kerala state assembly elections 2016, one month before the polling date. Political parties engaged in campaigns cancelled their mass campaigns for the day and involved in relief operations. The incident attracted wide attention in the country and captured the heat of state assembly election. The Prime Minister of India and a couple of national level political leaders having security cover of special protection group (SPG) visited the affected area and mass casualty managing hospitals within 16 hours of the explosion. Police engaged in emergency response and preparation of ‘mahazar’ and ‘inquest’ had to mobilize additional human resource and vehicles for VVIP (Very Very Important Person) security that otherwise could have been utilized for emergency management. The medical care facility of sterilized burn treatment divisions in hospitals was also defiled by the VVIP visits accompanied by the entourage and local politicians, since the seriously burn injured are highly vulnerable to infections. Disaster politicization and VVIP visits during the critical hours of emergency response may hamper the response and relief operations. The national disaster management policy and plan do not contain a protocol system to discourage impulsive VVIP visits to the disaster location that divert resources or delay prompt response operations.

## Phase III: Victim Support and Post Event Response

The incident took the lives of 109 people and 1250 individuals were injured. An ex-gratia of USD 14700 for the deceased, USD 2950 for the seriously injured and USD 750 for minor injuries were granted by the State Government. A medical board constituted by the state health department examined the injured cases and categorized the injuries as major and minor. The injured were also treated in public and private hospitals at the expense of the State Government.

As per the provisions laid down by the 14th Finance commission for the operation of disaster response fund^4^ , the State executive committee of Kerala State Disaster Management Authority declared the incident as ‘state specific disaster’ so as to facilitate financial assistance to the affected victims from State Disaster Response Fund. The ex-gratia compensation allotted by the State Government to the kin of the victims of this disaster would be the highest relief offered in a human induced disaster in India. The Union Government also declared an ex-gratia of 2950 USD to the deceased and USD 750 for seriously injured. In addition to that State Government released immediate relief assistance of USD 150/- to hospitalized victims in three days of time. The cabinet sub-committee of the State Government reviewed relief operations, visited the disaster site and assessed infrastructure damages on 14.04.2016 to approach the Union Government at New Delhi for a rehabilitation package. A memorandum was submitted to the Union Government demanding that the event be declare as a ‘National Disaster’ and requested financial assistance of USD 17.54 million for the long term recovery of the affected area. The ministry of home affairs has cleared their views on the memorandum submitted by the Government of Kerala in such as way that the exiting guidelines do not contemplate declaring incident as national calamity and hence further assistance was not released by the Government of India^5^ for long-term recovery.

## Fireworks: Safety and Preparedness

In the light of the above description, it is essential to trace back the fireworks safety mechanism in India and an appraisal of its execution in Puttingal temple fireworks. Every year, the competitive fireworks at Puttingal Temple had been damaged doors, windows, electrical appliances and even sheared walls of houses located within 200 meter radius of the firing point. The community residing around the temple accepts the minor economic losses incurred from the fireworks as ‘routine risk’ revisiting annually. A risk adaptation practice followed by the residents to reduce the impact of blast waves is that they covered glassed windows and doors with insulation tapes and kept them open during the firework hours. The local community admitted the routine risk upon religious sentiments raised by the firework lovers. But the routine risk was replaced by ‘rarest of the rare disaster’ on 10.04.2016.

The Explosives Act of 1884 and explosive rules 2008, regulate the manufacturing, possession, transport, sale and use of explosives in India. The organizer of a public staging event has to apply for permission in form no LE.6 (Explosives Act, 1884) adhering to the strict conditions that stipulated in explosive rules 2008. A deviation from the conditions specified to put into practice for public fireworks safety, the rules entrust the district magistrate to deny permission for staging the display. The mandatory safety distance shall be maintained between firing point and the spectator crowd is 100 meter^6^ . A safe distance of 50 meter shall also be maintained between firecracker store house and firing point. The authors has observed that Puttingal temple and majority of other religious public firework sites located in the urban as well as rural areas in Kerala does not meet the norms of legal requirements as enacted in India after 1980s.

Request of the Puttingal Devi Temple administration to hold fireworks had been denied by the district administration on the basis of field inspection reports from Police, Fire and Rescue, Pollution Control Board, Tahsildar and a petition filed by a neighboring resident of temple. The temple had several limitations on its infrastructure and space availability to meet the norms of public firework displays. Minimum distance between the crowd and firework point could not have been maintained as several houses are located within 100 meter radius. However, the fire work was staged despite the repudiation of district administration to permit the firework display. The rule of law was absolutely violated and the firework was displayed on the nexus of religious denominations with political overlords and a part of general public. Hence, the incident can be called as ‘socially constructed disaster’ that resulted from the culmination of negligence of law, non-sensitivity of public towards safety, competitive nature of event organizers, exploitation of self interests of a sect of people on the cover of religious sentiments and finally the failure of authorities in executing the legal orders passed.

## Conclusion

The operationalization of firework public display in India is in a fragmented state and requires urgent attention to avoid disasters in the short and long run. The ‘foreman’s certificate’ is issued by the Controller of Explosive to a person who is conversant with safe manufacture, storage, transportation, handling of explosives. Explosive rules have not made it clear as to what kind of educational qualifications and training must be possessed by a person before he makes an application for ‘foreman’s certificate’. The basic chemistry of explosives and professional training on firecracker management is essential to enable a person engaging with public display of fireworks. Individuals holding foreman’s certificate have not undergone any kind of professional training or certification; rather they learned the manufacturing and usage from their predecessors or traditional groups. Each event of public fireworks attracts thousands of people. The most popular annual firework in Kerala, the ‘Thrissur Pooram’ is attended by more than hundred thousand people where the licensee employs hundreds of workers to stage the display. A mishandling or a deviation from any of the safety procedures would be disastrous to the entire crowd. Hence, professional training and certification has to be brought in to make the sector safe and scientific.

Event safety and crowd safety are still at nascent stage in the country and has not received due priority even after repeated disasters over religious mass gatherings. The firecracker disaster at Puttingal Goddess temple is one of the ‘rarest of the rare disaster’ occurred in India. Human loss and economic loss incurred from the disaster is higher than the occasional firecracker explosions reported from manufacturing units or festival venues. The public display of fireworks was conducted without official permission and not following mandatory safety requirements. The incident raises a question on risk regulatory mechanisms and compliance. Event organizers and regional community pressure channelized the risk accumulation to the unlicensed firework event. The government machinery was awakened by the explosion that facilitated the response operations in a most efficient and timely manner with the available resources. That the disaster struck is a clear example of the lack of enforcement of existing regulations or festival mismanagement that disturbs the safety concerns of a community with the power of religious sentiments. The traditions and culture imposing potential risk on the community need to be revisited on safety facet. Event safety is yet to occupy its prime position in the festival management system. Enforcement agencies can utilize community involvement and public intelligence to overcome the demands. Public and event organizers need to be sensitized about the safety regulations and impacts of fireworks. Above all, stringent enforcement and compliance of enacted rule of law is of prime importance to have safe firework events and to make public gatherings safe.

## Funding

The authors received no specific funding for this work.

## Competing Interests

The authors have declared that no competing interests exists under the PLOS Journal Policy.

## Data Availability

All data generated or analysed during this study are included in this article. Selected photographs taken by the authors during the field visit is available at https://figshare.com/articles/Firecracker_Explosion/5472382.

## Corresponding Author

Faisel T Illiyas

Tata Institute of Social Sciences

Mumbai, India

faiselses@gmail.com, faisel.illiyas2014@tiss.edu

+91 9447203981, +918333905024
